# Transient basilar artery occlusion monitored by transcranial color Doppler presenting with a spectacular shrinking deficit: a case report

**DOI:** 10.1186/1752-1947-4-13

**Published:** 2010-01-19

**Authors:** Giuseppe Nicoletti, Gerardina Albano, Sandro Sanguigni, Salvatore Tardi, Giovanni Malferrari, Massimo Del Sette, Filomena Bruno, Aldo Nicolai

**Affiliations:** 1 Geriatric Department, Madonna delle Grazie Hospital, via Cattedra Ambulante 75100 Matera, Italy; 2 Neurology Department, Madonna delle Grazie Hospital, Via Cattedra Ambulante, 75100 Matera, Italy; 3 Neurology Department, Madonna del Soccorso Hospital, S Benedetto del Tronto, Italy; 4 Neurology Department, Santa Maria Nuova Hospital, Reggio Emilia, Italy; 5 Department of Neurosciences, Ophthalmology and Genetics, University of Genoa, Genoa, Italy

## Abstract

**Introduction:**

We describe the case of a 79-year-old Caucasian Italian woman with a transient basilar occlusion monitored by transcranial Doppler, with subsequent recanalization and clinical shrinking deficit. This is the first case of transient basilar occlusive disease diagnosed and monitored by transcranial Doppler. This case is important and needs to be reported because transient basilar occlusion may be easily diagnosed if transcranial Doppler is performed.

**Case presentation:**

A 79-year-old woman affected by chronic atrial fibrillation and not treated with oral anticoagulants, cardioverted to sinus rhythm during a gastric endoscopy. She then showed a sudden-onset loss of consciousness, horizontal and vertical gaze palsy, tetraparesis and bilateral miosis and coma. Two hours later, the symptoms resolved quickly, leaving no residual neurologic deficits. Transcranial Doppler examination showed a dampened flow in the basilar artery in the emergency examination and a restored flow when the symptoms resolved.

**Conclusion:**

This is the first case of transient basilar occlusive disease diagnosed and monitored by transcranial Doppler. We believe that transcranial Doppler should be performed in all cases of unexplained acute loss of consciousness, in particular, if associated with signs of brainstem dysfunctions.

## Introduction

Embolic occlusion of the basilar artery has been described as a dramatic event, with a severe and even fatal outcome if occlusion is permanent, and with a more benign course if the occlusion is transient [1]. Nevertheless, there are few cases where the clinical course has been correlated with basilar artery flow monitoring. In fact, the diagnosis of embolism in the basilar artery is often difficult; in some patients, symptoms resolve quickly, leaving no residual neurologic signs, and neuroradiologic findings may also be unremarkable.

We describe the case of a 79-year-old woman with a typical clinical syndrome of basilar occlusion, in whom contrast-enhanced transcranial Doppler demonstrated the presence of a basilar occlusion that suddenly resolved, with parallel resolution of symptoms.

## Case presentation

**Figure 1:**
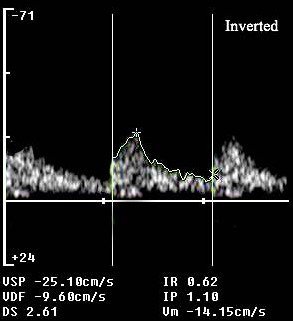
**On patient presentation, the transcranial Doppler showed a dampened flow at the level of the basilar artery: a pulsatile flow with normal flow acceleration and decreased mean flow velocity (>30% difference between the proximal and distal arterial segments)**.

A 79-year-old Caucasian Italian woman affected by chronic atrial fibrillation and not treated with oral anticoagulants, cardioverted to sinus rhythm during a gastric endoscopy; she then showed a sudden onset loss of consciousness. She was admitted to the emergency department, where pO_2 _saturation, arterial blood gases analysis, electrocardiogram and laboratory testing were all found to be normal. Neurological examination showed coma, horizontal and vertical gaze palsy, tetraparesis and bilateral miosis. Non-enhanced computed tomographic scanning of her head was unremarkable. The patient was then admitted to the neuro-geriatric ward, where an emergency complete ultrasound examination was performed. An extracranial duplex sonography of the carotid and vertebral arteries was unremarkable. A transcranial Doppler with suboccipital approach showed the typical pattern of 'dampened flow' (mean flow velocity 14.1 cm/sec; resistance index (RI) 0.62; pulsatility index (PI) 1.10) on the basilar artery at 75 mm depth [2,3] (Figure 1). The dampened flow is a typical sign of recanalization in the case of intracranial artery occlusion [3,4]. A transcranial Doppler with transtemporal approach showed normal findings on middle, anterior and posterior cerebral arteries bilaterally. The flow in the posterior cerebral arteries was normal because there was activation of the posterior communicating arteries.

Low-molecular-weight heparin at therapeutic dosage (enoxaparin 1 mg/kg subcutaneously twice daily) was started immediately. Two hours later, the symptoms resolved quickly, leaving no residual neurologic deficits.

**Figure 2:**
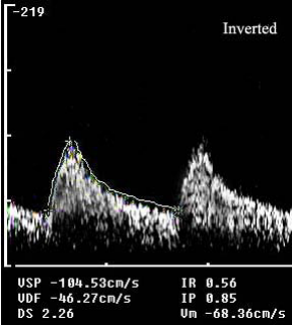
**Two hours later, the transcranial Doppler showed a stenotic flow at the level of the basilar artery: a focal mean flow velocity increase >30% compared with the proximal arterial segment**.

**Figure 3:**
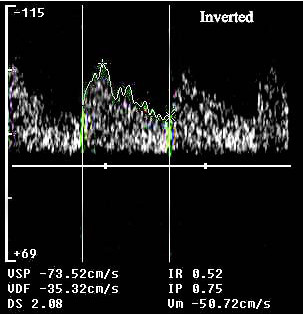
**On the following day, the transcranial Doppler showed a normal flow at the level of the basilar artery: a low-resistance antegrade flow with mean flow velocity <60 cm/sec**.

A control transcranial Doppler showed restored flow with a minor velocity increase at the level of the proximal basilar artery (mean flow velocity: 68.3 cm/sec), probably an expression of residual stenosis (Figure 2). On the following day, the patient underwent magnetic and angiomagnetic resonance imaging, which did not show any parenchymal or vessel abnormality. Then a transcranial Doppler was performed and normal blood flow velocities were documented (Figure 3).

## Discussion

We report a case of spectacular shrinking deficit [4] in a patient with basilar occlusion, probably due to cardioembolism, documented by transcranial Doppler and followed by vessel recanalization.

The diagnosis of basilar embolism and occlusion is difficult in patients with brainstem syndromes with complete recovery and with no radiologic evidence of infarctions. The most common causes of basilar artery occlusion are emboli that arise from the heart, while atherosclerosis of the aorta and the vertebral artery, arterial dissection, patent foramen ovale, angiographic or vascular surgical complications are less frequent causes [5,6].

Deterioration of consciousness, tetraparesis, hemiparesis, ataxia, pupillary abnormalities, gaze palsy, and cortical blindness are frequent symptoms in patients with basilar artery occlusive disease [1,5,6]. Transient basilar occlusion can resolve quickly leaving no residual neurologic signs or neuroradiologic abnormalities, while rarely, transient basilar artery occlusion has a poor outcome if fragments of an embolus cause bilateral brainstem infarcts or large cerebellar infarctions [1].

In our patient, the shrinking deficit occurred without systemic or local therapeutic thrombolysis, probably due to an intrinsic fibrinolytic mechanism, possibly helped by heparin treatment. As the embolus probably dissolved quickly into smaller fragments, it caused only transient symptoms, with no evidence of cerebral damage on magnetic resonance imaging. The evidence of basilar occlusion by transcranial Doppler confirmed the vascular origin of the clinical syndrome. The temporal relationship with cardioversion suggested the cardio-embolic origin of the episode.

As the diagnosis of basilar embolism is difficult in patients in whom symptoms resolve quickly and neuroradiologic findings are negative, many cases of embolism in the basilar artery probably remain undiagnosed or are incorrectly diagnosed. In patients with acute brain stem symptoms and loss of consciousness, a rapid assessment of blood flow through the basilar artery is crucial. Digital subtraction angiography is the gold standard for evaluation of patients with clinically suspected acute basilar artery occlusion. However, this is an invasive, costly and time-consuming procedure associated with a small risk of complications [7]. In our patient, the deficit resolved spontaneously in a few hours, thus we did not perform diagnostic or therapeutic digital subtraction angiography. Transcranial Doppler was chosen above traditional methods because it is non-invasive and low-cost; furthermore, it allows study of intracranial hemodynamics at the patient's bedside. Using the well-established grading system for diagnosis of residual flow in brain ischemia [3], we could identify the presence of Thrombolysis in Brain Ischemia (TIBI) 3 score in the acute phase (Figure 1), which turned into stenotic flow (TIBI 4) in 2 hours (Figure 2).

Initially, transient occlusion of the basilar artery may occur with sudden loss of consciousness as the only symptom, possibly followed by deficits and brainstem dysfunctions. While permanent basilar artery occlusion is a severe disease with poor outcome, transient basilar artery occlusion often has a benign outcome, and thus possibly is underdiagnosed. In our patient, transcranial Doppler was useful not only for diagnosis, but also for monitoring and for prognostic information.

## Conclusion

This is the first case of transient basilar occlusive disease diagnosed and monitored by transcranial Doppler. We think that transcranial Doppler should be performed in all cases of unexplained acute loss of consciousness, in particular, if associated with signs of brainstem dysfunctions.

## Abbreviations

PI: pulsatility index; RI: resistance index; TIBI: thrombolysis in brain ischemia.

## Consent

Written informed consent was obtained from the patient for publication of this case report and any accompanying images. A copy of the written consent is available for review by the Editor-in-Chief of this journal.

## Competing interests

The authors declare that they have no competing interests.

## Authors' contributions

GN performed the transcranial Doppler, and was a major contributor in writing the manuscript. AN, GA, SS, ST, MS revised the manuscript for important intellectual content. FB made substantial contribution to acquisition of data. ST has given final approval of the version to be published.

## References

[B1] Schwarz S Egelhof T Schwab S Hacke W Basilar artery embolism. Clinical syndrome and neuroradiologic patterns in patients without permanent occlusion of the basilar artery Neurology 1997 49 1346 1352 9371920 10.1212/wnl.49.5.1346

[B2] Demchuk AM Christou I Wein TH Felberg RA Malkoff M Grotta JC Alexandrov A Specific transcranial Doppler flow findings related to the presence and site of arterial occlusion Stroke 2000 31 140 146 10625729 10.1161/01.str.31.1.140

[B3] Demchuk AM Scott Burgin W Christou I Felberg RA Barber P Hill MD Alexandrov A Thrombolysis in brain ischemia (TIBI). Transcranial Doppler flow grades predict clinical severity, early recovery, and mortality in patients treated with intravenous tissue plasminogen activator Stroke 2001 32 89 93 11136920 10.1161/01.str.32.1.89

[B4] Minematsu K Yamaguchi T Omae T 'Spectacular shrinking deficit': rapid recovery from a major hemispheric syndrome by migration of an embolus Neurology 1992 42 1 157 162 1734297 10.1212/wnl.42.1.157

[B5] Caplan LR Wityk RJ Glass TA Tapia J Pazdera L Chang HM Teal P Dashe JF Chaves CJ Breen JC Vemmos K Amarenco P Tettenborn B Leary M Estol C Dewitt LD Pessin MS New England Medical Center Posterior Circulation registry Ann Neurol 2004 56 389 398 10.1002/ana.20204 15349866

[B6] Caplan LR Posterior Circulation Disease: Clinical Findings, Diagnosis, and Management 1996 Cambridge, Mass: Blackwell Science

[B7] Dawkins AA Evans AL Wattam J Romanowsky CA Connolly DJ Hodgson TJ Coley SC Complications of cerebral angiography: a prospective analysis of 2924 consecutive procedures Neuroradiology 2007 49 9 753 759 10.1007/s00234-007-0252-y 17594083

